# The Dynamics of Wealth Inequality and the Effect of Income Distribution

**DOI:** 10.1371/journal.pone.0154196

**Published:** 2016-04-22

**Authors:** Yonatan Berman, Eshel Ben-Jacob, Yoash Shapira

**Affiliations:** School of Physics and Astronomy, Tel-Aviv University, Tel-Aviv, Israel; University College London, UNITED KINGDOM

## Abstract

The rapid increase of wealth inequality in the past few decades is one of the most disturbing social and economic issues of our time. Studying its origin and underlying mechanisms is essential for policy aiming to control and even reverse this trend. In that context, controlling the distribution of income, using income tax or other macroeconomic policy instruments, is generally perceived as effective for regulating the wealth distribution. We provide a theoretical tool, based on the realistic modeling of wealth inequality dynamics, to describe the effects of personal savings and income distribution on wealth inequality. Our theoretical approach incorporates coupled equations, solved using iterated maps to model the dynamics of wealth and income inequality. Notably, using the appropriate historical parameter values we were able to capture the historical dynamics of wealth inequality in the United States during the course of the 20th century. It is found that the effect of personal savings on wealth inequality is substantial, and its major decrease in the past 30 years can be associated with the current wealth inequality surge. In addition, the effect of increasing income tax, though naturally contributing to lowering income inequality, might contribute to a mild increase in wealth inequality and vice versa. Plausible changes in income tax are found to have an insignificant effect on wealth inequality, in practice. In addition, controlling the income inequality, by progressive taxation, for example, is found to have a very small effect on wealth inequality in the short run. The results imply, therefore, that controlling income inequality is an impractical tool for regulating wealth inequality.

## Introduction

The surge in wealth inequality is one of the most disturbing social and economic issues of our time. The rapid increase in wealth inequality has generated much effort to understand the origin and possible control of this trend [1–7]. For a comprehensive review of historical theories and analyses of wealth inequality please refer to [6]. Wealth inequality is generally thought to impose instabilities on economies and on the social structure of countries [6, 8–10]. In most western countries, in which wealth inequality had dramatically increased during the past 30 years, personal savings had substantially decreased [7, 11]. This decrease was found to be one of the major origins of the recent surge in inequality [7, 12–14]. As a consequence, it might be possible to apply an economic policy leading to the reduction of wealth inequality in the future.

**Fig 1:**
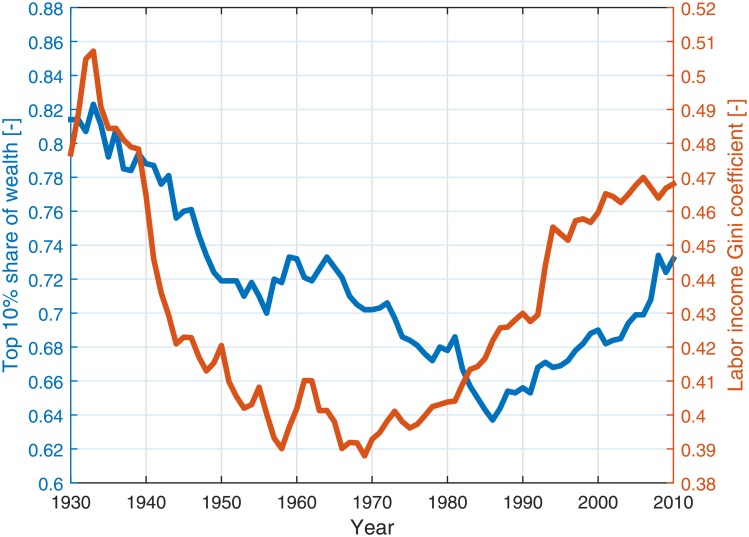
Historical economic inequality in the US. The Wealth (blue) and labor income (orange) inequality (pre-taxes) in the US for 1930–2010. The wealth inequality is quantified by the share of wealth owned by the richest 10% of the population. The income inequality is measured using the Gini index [19]. Data is taken from Piketty and Saez [3] and Saez and Zucman [20].

In addition to wealth inequality, income inequality had increased dramatically in the past few decades in many countries. These two types of inequality are closely related and are positively correlated, as labor income is a major source of wealth. Fig 1 depicts the historical trend of wealth and income inequality in the US during the past 8 decades. However, despite the relation between these notions they still differ considerably. In several developed countries, such as Denmark and Switzerland, income inequality is very low, while the wealth inequality is among the highest in the western world [15–17]. The differentiation between income and wealth inequality is important, since affecting one might not necessarily affect the other and vice versa. It is also important due to the confusion found at times between them in the media, and even within the scientific community. Most of the research devoted to economic inequality is focused on income inequality. It is usually easier to measure, and garnered massive media coverage and public attention in the past few years. We focus, however, on wealth inequality, as it reflects better the real social and economic gaps within a society [18].

Wealth inequality is always higher than labor income inequality, due to income originated from wealth (or capital income), such as rents, dividends or royalties and the increase of asset values [6]. This source of income accounts for a large portion of the national income (≈ 20%−35% [11]), hence its substantial effect on wealth inequality. Additionally, though labor income accounts for most of the national income produced in all western economies, only a relatively small fraction of it contributes to accumulating personal wealth due to taxation and spending.

The specified differences between wealth and income inequalities join the imperfect correlation between wealth and income found in the US [21–23], of about 0.55–0.6 [18, 21, 23, 24]. This correlation implies that the effect of controlling the income distribution on the wealth distribution is limited. It also suggests that the recent wealth inequality surge is only partially related to the income inequality surge, both depicted in Fig 1.

In order to determine the underlying mechanisms affecting wealth inequality, a quantitative analysis is needed. We need to identify which of the factors that affect inequality play a major role and which a secondary role. Such an analysis will provide necessary information for policy making aiming to immediately suppress the soaring rate of wealth inequality and even further reduce it in the future. In particular, we wish to estimate the effect of income tax and income inequality on wealth inequality. Regardless of the imperfect correlation observed between income and wealth, it had been suggested that “progressive taxation can powerfully affect income and wealth concentration” [20, 25]. The immediate effect of income tax on income inequality is rather clear, however, its effect on the distribution of wealth is intricate and requires proper modeling.

Agent-based or individual-driven models for the dynamics of wealth accumulation have been extensively studied recently [7, 12, 26–35]. Some of the models consider a strong interaction between agents within the population when addressing inequality, while in other model types wealth exchange between individuals is incorporated in order to provide theoretical insights on the shape of the wealth distribution, inspired by kinetic processes in gases. Other models emphasize the importance of intergenerational elasticity and inheritance. Most of the proposed models consider the multiplicative nature of wealth accumulation and treat wealth as a stochastic process [7, 34, 36].

Here, we present a new model devised to study the dynamics of the wealth inequality, solved using multiple iterated maps. We incorporate in the model published information regarding the personal wealth, disposable income, personal savings and income inequality as the parameters that govern these dynamics. The model provides a theoretical framework to quantify the contributions of the factors affecting the wealth inequality and offers a valuable test-bed for predicting the effect of various policies on wealth inequality. It is loosely related to the model presented in [7], however, it includes substantial changes, refinements and improvements.

By implementing the model using a numerical simulation and considering the historical data for the different parameters in the US economy from 1930 to 2010, we compared and found an excellent agreement between the model and the historical dynamics of wealth inequality over this long period of time. The model can therefore be used as a predictor too, thus contributing to the analysis of the relative effect of the various parameters on future wealth inequality. It can be used to test which of the mechanisms that govern the dynamics of wealth inequality is primarily associated with the recent surge. Such an analysis could also provide insights on processes which might lead to the reduction of wealth inequality. Although the analysis was mainly done for the US economy, we present its general implications and discuss the validity of the results for different types of economies.

## Model

We devise a model for the dynamics of wealth inequality. The model describes wealth accumulation of individuals within a population from 3 sources:

1. Labor income—accounts for the income originated from wages and earnings. Only a fraction of the labor income contributes to the accumulated wealth, due to taxation and spending.
2. Capital income—accounts for the income originated from wealth, including profits, royalties, rents and dividends. Only a fraction of it contributes to the accumulated wealth, due to taxation and spending. Capital and labor income together constitutes the total national income.
3. Capital value change—accounts for the value change of capital such as owned land, shares, options and other assets owned by individuals. Some of the capital income, a part of the national income, is invested back, contributing to an increasing value of assets, accounted therefore as capital value change and not as capital income.

The contribution of these sources to wealth can be formulated using a simple differential equation:

$$\begin{array}{c} \frac{dW\left(t\right)}{dt}=\alpha \left(t\right)W\left(t\right)+\sigma \left(t\right)D\left(t\right)\;, \end{array}$$ (1)

where *W*(*t*) is the wealth, *α*(*t*) is the capital value change rate, *D*(*t*) is the total disposable income and *σ*(*t*) is the rate in which the total disposable income is saved.

In order to solve Eq (1), we consider a population of *N* individuals, in which each individual *i* is characterized at discrete time steps *n*, by the wealth it owns *W*_*i*_(*n*) and by its disposable income *D*_*i*_(*n*). The time interval between consecutive time steps is taken as one year, due to the availability of data.

The distributions from which the initial wealth and income of each individual are drawn were taken from Piketty and Zucman [11] for the income distribution and from Wolff [37] for the wealth distribution. These distributions reflect the actual personal wealth and disposable income distributions in the US at 1930. Each individual starts with a randomly chosen value of income and wealth, and we make sure that the sample wealth and income initial distributions are created so that a correlation of 0.55 between them is maintained.

The obtained correlation between wealth and income changes in time, however this change is very small (±2%), which agrees with its historical values [21, 23], and indicates that the changes in the wealth and income distributions are relatively mild. This result (depicted graphically in S1 Fig) hints that the changes in the distributions are mainly due to small changes in individual wealth and income values, rather than due to mobility (see also the following sections).

**Fig 2:**
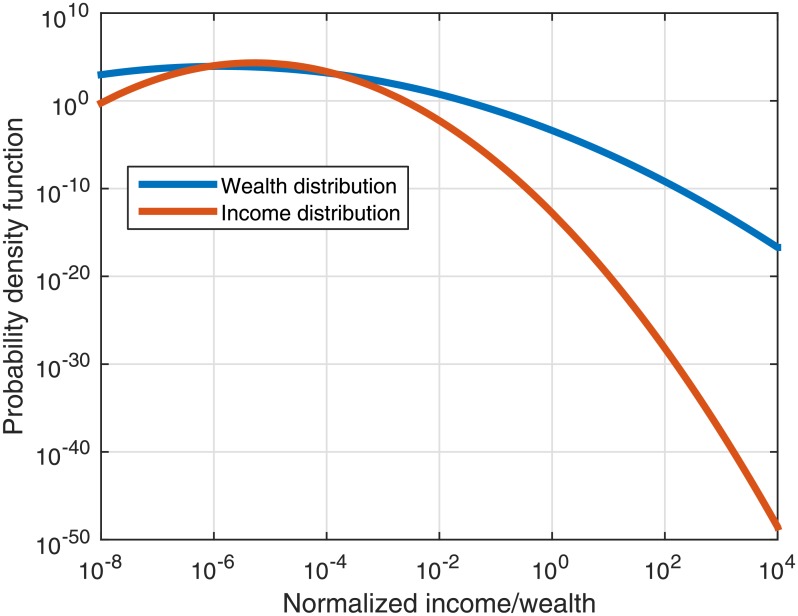
The initial distributions of income and wealth. The Wealth (blue) and disposable income (orange) distributions representing the distributions from which the initial individual wealth and income values are drawn in the model. The wealth and income were normalized for proper display. These distributions depict the realistic 1930 wealth and income distributions in the US. The data are taken from Piketty and Zucman [11] for the income distribution and from Wolff [37] for the wealth distribution.

The wealth and income initial distributions are presented in Fig 2.

Let us denote $W(n)=\sum_{i=1}^{N}W_{i}(n)$, as the total personal wealth and $D(n)=\sum_{i=1}^{N}D_{i}(n)$ as the total disposable income at time step *n*. Following these notations, the total personal wealth at time *n* + 1 is equal to the total personal wealth at time *n* added to the capital value change, denoted as *A*(*n*), and to the income contribution to wealth. This contribution is calculated by taking into account the average personal savings rate from disposable income *s*(*n*). This equality can be put into the following discrete equation (consistent with Eq (1), but not immediately derived from it):

$$\begin{array}{c} W\left(n+1\right)=W\left(n\right)+A\left(n\right)+s\left(n\right)D\left(n\right)\;. \end{array}$$ (2)

The historical values of all variables in Eq (2) can be extracted from various sources [11, 38, 39], apart from *A*(*n*), which is calculated simply by solving the equation using the extracted data. We use the obtained data and define $a(n)=\frac{A(n)}{W(n)}$ as the capital value change rate.

The integral data obtained by extracting the historical data and solving Eq (2), can be now used as input of an equation describing the accumulation of wealth for a single individual. Solving this equation for all individuals will enable us to calculate the distribution of wealth at each time step, hence the wealth inequality.

The dependence of savings on income is due to the tendency of individuals with higher income to save a larger fraction of it [40–45]. We assume this dependence is constant in time, though in fact it changed through history. However, the model results are not very sensitive to such changes (see following sections). In order to consider this dependence, we divide the population to income deciles, and multiply the income of each individual by a corresponding factor $\chi_{i}^{D}(n)$, which satisfies $\sum_{i=1}^{N}\chi_{i}^{D}(n)D_{i}(n)=D(n)$ and hence also:

$$\begin{array}{c} \sum_{i=1}^{N}s\left(n\right)\chi_{i}^{D}\left(n\right)D_{i}\left(n\right)=s\left(n\right)D\left(n\right)\;. \end{array}$$ (3)

Additionally, we consider the dependence of capital value change on wealth, divide the population to wealth deciles, and multiply the wealth of each individual by a corresponding factor $\chi_{i}^{W}(n)$, which satisfies $\sum_{i=1}^{N}\chi_{i}^{W}(n)W_{i}(n)=W(n)$ and hence also:

$$\begin{array}{c} \sum_{i=1}^{N}a\left(n\right)\chi_{i}^{W}\left(n\right)W_{i}\left(n\right)=a\left(n\right)W\left(n\right)=A\left(n\right)\;. \end{array}$$ (4)

**Fig 3:**
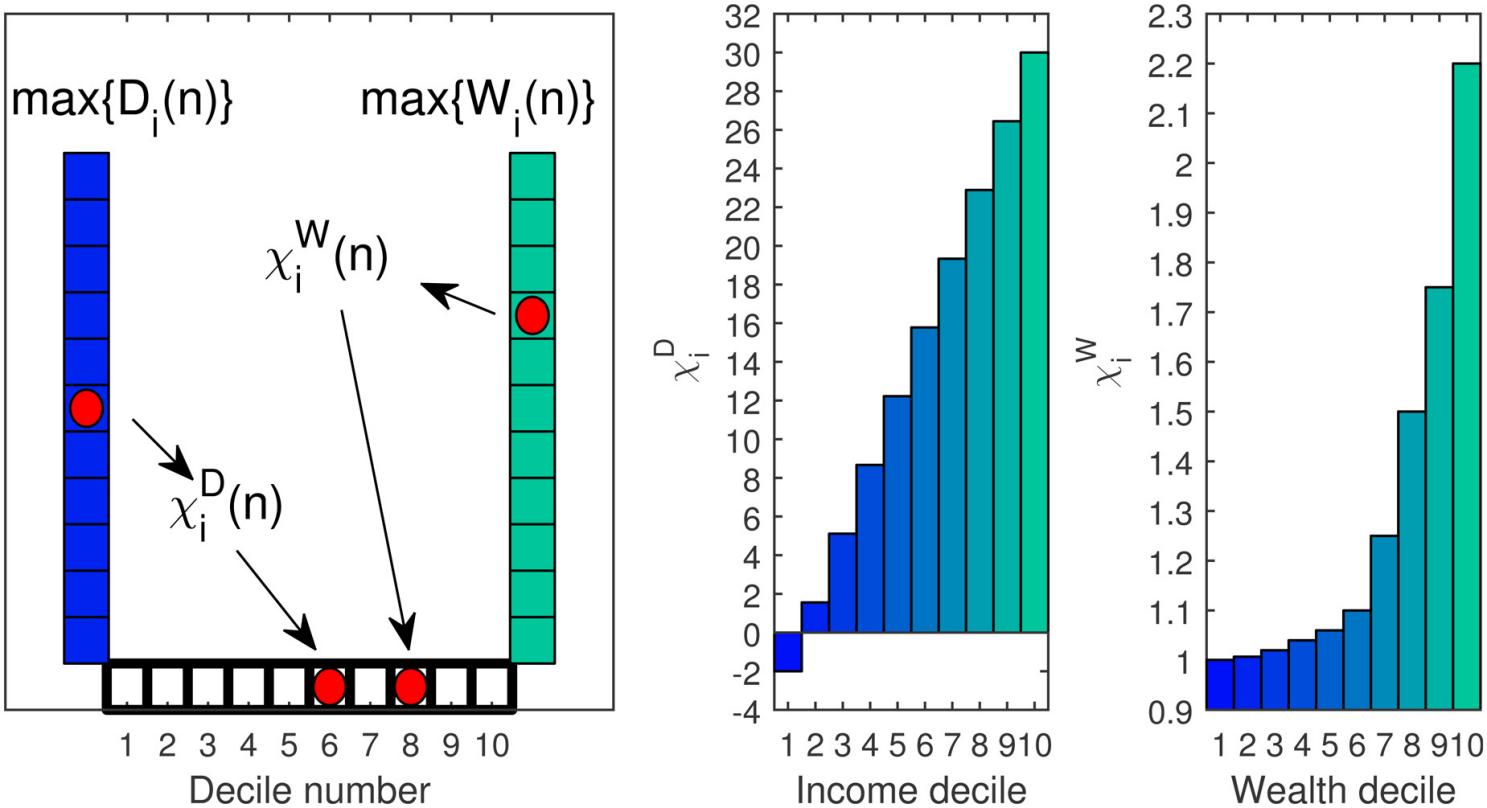
The division of wealth and income to deciles. Left panel: An illustration of the division to deciles—every time step *n*, each individual, with disposable income *D*_*i*_ and wealth *W*_*i*_ is attached to a certain decile in income and wealth according to the distribution of the entire population. Middle panel: The dependence of the disposable income fraction, $\chi_{i}^{D}$, on the income decile. Right panel: The dependence of $\chi_{i}^{W}$ on the wealth decile. The presented values of $\chi_{i}^{D}$ and $\chi_{i}^{W}$ are not normalized. In practice the values are normalized so that $\sum_{i=1}^{N}s(n)\;\chi_{i}^{D}(n)D_{i}(n)=s(n)D(n)$ and $\sum_{i=1}^{N}a(n)\;\chi_{i}^{W}(n)W_{i}(n)=a(n)W(n)$.

The dependence of $\chi_{i}^{W}$ on wealth is mainly due to different asset classes owned by wealthier individuals compared to poorer individuals [20, 46], in addition to better terms of investment and investment possibilities accessible to rich individuals, but inaccessible to the poor [6, 9, 47]. We assume this dependence is constant in time and is constructed by the data provided by Wolff [46], and set the highest value of $\chi_{i}^{W}$ to be 2.2 times more than the lowest value. The model results are not very sensitive to this choice (see S2 Fig for additional information), however, if no dependence is taken into account (meaning that $\chi_{i}^{W}=\chi^{W},\;\;\forall i$), no increase in wealth inequality can be possible, based on capital accumulation. In such case, the share of wealth owned by the top deciles will not increase and will only decrease or stay unchanged. A typical dependence of $\chi_{i}^{D}$ and $\chi_{i}^{W}$ on income and wealth is depicted in Fig 3.

Based on the above and on Eq (2), we can formulate an equation for the wealth accumulation of each individual *i*:

$$\begin{array}{c} W_{i}\left(n+1\right)=W_{i}\left(n\right)+\chi_{i}^{W}\left(n\right)a\left(n\right)W_{i}\left(n\right)+\chi_{i}^{D}\left(n\right)s\left(n\right)D_{i}\left(n\right)\;. \end{array}$$ (5)

Eq (5) is clearly consistent with Eq (2), meaning that summation over *W*_*i*_(*n* + 1) for all individuals results in Eq (2). By solving the iterated map for all individuals, we obtain the wealth distribution at each time step.

We note that it is also essential to properly propagate the disposable income in time, done using the following relation:

$$\begin{array}{c} D_{i}\left(n+1\right)=D_{i}\left(n\right)\frac{D\left(n+1\right)}{D\left(n\right)}\;, \end{array}$$ (6)

where *D*(*n* + 1) and *D*(*n*) are taken from published historical data.

Following the obtained wealth distribution, we calculate the share of wealth owned by the top wealth decile. This measure will be used as the primary measure for wealth inequality within the scope of this work.

An additional parameter in the model, which is not a part of Eqs (2–5) is the income distribution, measured by its Gini index. Each time step, before the propagation of wealth and income according to Eq (5), we redistribute the income so that the actual historical value of the income Gini index, denoted as *G*(*n*) is maintained. In order to perform this redistribution, we multiply the income of the individuals that belong to each income decile *i* by a factor close to 1, *ϵ*_*i*_, where *i* = 1, 2, …, 10. The values of *ϵ*_*i*_ are calculated every time step using the simplex search method [48]. The value of these factors lie within the interval (0.96,1.04) with a mean of 1 and standard deviation of 1%. As previously stated, after this calculation, we also make sure that the total income is maintained. The historical data for the income Gini index in the US are taken from [3]. We note that the after-tax Gini index is used, since we are interested in the distribution of the disposable income. The model results are not very sensitive to the value of *G*(*n*), and assuming a constant characteristic value provides a good approximation for the actual results (see S3 Fig for additional information). In order to take into account the effect changing the income inequality has on wealth inequality, the described procedure will be applied in the following calculations.

**Fig 4:**
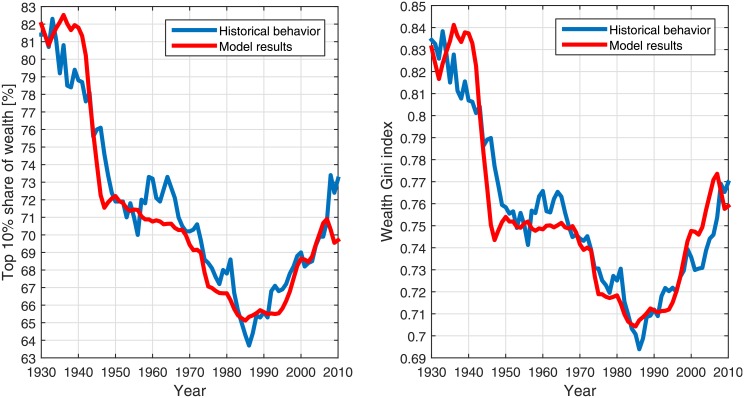
The model results for the historical wealth inequality measures in the United States during 1930–2010. The results produced by implementing the model (red) were calculated using the historical data for the various parameters [11, 20]. The historical data (blue) were taken from Saez and Zucman [20]. The results are given for the top 10% share of wealth (left) and for the reconstructed historical wealth Gini index (right), based on Saez and Zucman [20].

A comparison between the model results and 8 decades of the wealth inequality dynamics in the US is shown in Fig 4. More specifically, we show a comparison with the wealth owned by the top decile in the US in the period 1930–2010 [20]. The results indicate the existence of a very high correlation (*ρ* = 0.96) between the modeled simulations and the historical data. This agreement is important in terms of the predictive power of the model. We note that specifically, in the end of the period in discussion (2009–2010), the model differs considerably from the historical behavior. This might indicate that the financial crisis of 2008 had unique characteristics relevant for the distribution of wealth, which are not taken into account in the model. In addition, it might indicate of inconsistencies in the wealth inequality data [20, 49] or in the personal savings data [11]. This will be further explored in a future work.

The choice in this measure and not the traditional Gini index for the quantification of wealth inequality is originated in the availability of data. The share of wealth of a certain fractile is easier to measure, while the wealth Gini index is only available for specific years and not for a long period of time. A reconstruction of the historical wealth distribution, from which the Gini index can be calculated, based on the fractile share of wealth data by Saez and Zucman [20] was done. However, the reconstruction process increases the uncertainty of the data. The historical reconstructed Gini along with the model results for it, are also presented in Fig 4. This result demonstrates that the model results for the top 10% wealth share and for the wealth Gini index are both in good agreement with the historical behavior. The correlation between the model results and the historical reconstructed Gini index was 0.94.

We note that due to the randomly distributed initial values of wealth and income, each run of the simulation produces slightly different results, given the same parameters. Therefore, a sufficient population size should be taken in order to obtain consistent results. We used a population of *N* = 10^7^ individuals, which provided an average difference of less than 0.01% in wealth inequality between two independent realizations of the simulation. This statistical spread translates into statistical error on the model results, which can be quantified. By running similar realizations of the model and calculating the spread, we obtained a very small statistical error, which is practically insignificant, if a large enough sample, such as *N* = 10^7^, is considered (see S4 Fig for additional information).

## The Model’s Predictive Power

Based on the good agreement of the model results with the historical behavior of wealth inequality, we wish to test its predictive power. In order to perform such a test, retrospective predictions and future predictions were done for the periods 1980–2010 and 2008–2030, respectively, as follows.

First, we calculated the wealth inequality dynamics using the historical values of the different parameters for 1930–1980 (1930–2007), as depicted in Fig 4. Second, we calculated the wealth inequality dynamics for the period 1980–2010 (2008–2030) given five sets of test parameters representing different policy scenarios defined below for the retrospective predictions (future predictions):

1. Unchanged parameters (Extrapolated parameter scenario)—The values of the parameters are taken as the averaged value of the parameters during the preceding period of 1970–1980 (The values of the parameters are linearly extrapolated during 2008–2030 based on their values during 2000–2007).
2. Decreasing savings scenario—The values of the parameters are taken as described in (1), except for the savings fraction that linearly decreases to -3% at 2010 (to -3% at 2030).
3. Increasing savings scenario—The values of the parameters are taken as described in (1), except for the savings fraction that was taken to be linearly increasing to 15% at 2010 (to 15% at 2030).
4. Decreasing income inequality—The values of the parameters are taken as described in (1), except for the income Gini index that linearly decreases to 0.25 (after tax) at 2010 (to 0.15 at 2030).
5. Increasing income inequality—The values of the parameters are taken as described in (1), except for the income Gini index that linearly increases to 0.5 (after tax) at 2010 (to 0.5 at 2030).

In addition, a simple linear extrapolation of the historical wealth inequality until 2030 was computed for comparison. These various scenarios can be interpreted as the outcomes of regulation and policy, changes in social structure and various other economic, political and social developments. Such changes and developments are impossible to predict, and most forecasts are given for only a few years ahead. The probability for each of the various scenarios to occur is not predictable. However, they form a diversified span of possible future outcomes.

**Fig 5:**
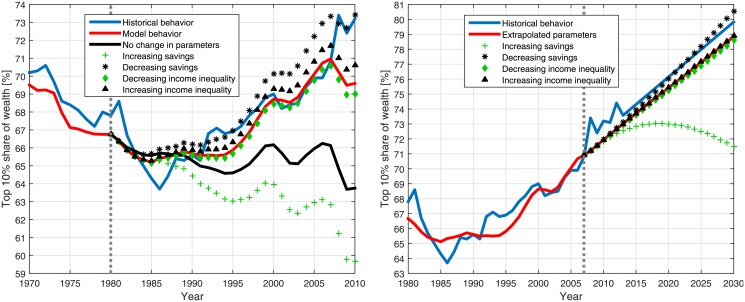
The predictive power of the model. Left panel: Retrospective predictions of the wealth inequality in the US. The blue and red curves present the historical wealth inequality behavior and the model behavior for the historical values of the parameters, respectively (see Fig 4). The results for the various scenarios during 1980–2010 are also presented: Unchanged parameter scenario (solid black curve), increasing savings scenario (green crosses), decreasing savings scenario (black stars), decreasing income inequality scenario (green diamonds) and increasing income inequality scenario (black triangles). The dotted gray line separates the calculation using historical parameter values and the retrospective prediction. Right panel: Future predictions of the wealth inequality in the US. The blue curve displays the historical wealth inequality behavior, in addition to a linear extrapolation for 2008–2030. The solid red curve presents the model results for the historical parameter values and the extrapolated parameter scenario. The results for the various parameter scenarios during 2008–2030 are also presented: increasing savings scenario (green crosses), decreasing savings scenario (black stars), decreasing income inequality scenario (green diamonds) and increasing income inequality scenario (black triangles). The dotted gray line separates the calculation using historical parameter values and the prediction for future inequality.

The results of these retrospective and future predictions are presented in Fig 5 and illustrate the predictive power of the devised model. The results imply that the model may be used to test the effect of future economic policies. As expected, if the parameter values are extrapolated, the wealth inequality is likely to increase at a similar pace as in 2000–2007. In addition, the results of the other scenarios demonstrate the substantial effect personal savings have on wealth inequality. In particular, if personal savings are significantly increased, wealth inequality is likely to dramatically decrease within the next few decades. This result is consistent with previous results [7]. The effect of increased or decreased income inequality is found to be relatively small.

## The Effects of Savings and Income Inequality

### The effect of personal savings on wealth inequality

**Fig 6:**
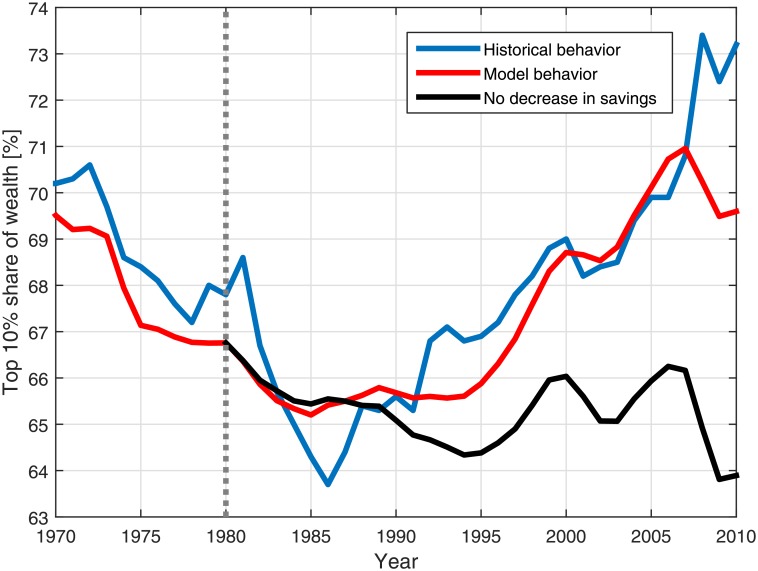
The effect of personal savings on wealth inequality. The blue and red curves present the historical wealth inequality behavior and the model behavior for the historical values of the parameters, respectively (see Fig 4). The black curve depicts the model results when the savings in the period 1980–2010 are taken as constant and equal to 10%, while the other model parameters are considered with their historical values. The dotted gray line separates between the calculation using historical parameter values and with the altered parameters.

The results in Fig 5 demonstrate that the savings rate is a principal parameter in the model. Increased savings lead to decreased wealth inequality, while decreased savings lead to increased wealth inequality. This observed effect of personal savings is well known [7, 12–14], and the results help to confirm that the inception of the recent inequality surge can be primarily associated with the decrease of personal savings [7]. In order to support this argument, a numerical simulation for the period 1980–2010 was performed, in which the savings fraction was taken as equal to 10%, a typical savings rate characterizing the early 1980s in the US. The rest of the parameters were taken with their historical values. The results are presented in Fig 6, and demonstrate that omitting the decrease in personal savings during 1980–2010 eliminates the surge in wealth inequality (compare with [7]).

The observed effect may seem counter intuitive due to the effect of the nonuniform distribution of savings, since it may seem that the rich use their money to earn larger returns and the poor have no savings with which they can produce returns or eliminate debt [50, 51]. However, the imperfect correlation between wealth and income enables the possibility of savings to reduce the relative gap between deciles. These results do not imply that extremely poor individuals can become very rich if they work and save a large fraction of their income. It only accounts for the possibility of mainly middle and higher wealth deciles to narrow the relative gap between their wealth and the wealth of the richer individuals. This is the effect observed in the above figures, driven by personal savings.

This can be demonstrated by the following example: Let us suppose that two individuals earn the same disposable income, *D*. One individual owns wealth of *W*_1_ and the other one owns *W*_2_ = *ρW*_1_, with *ρ* < 1. Since *W*_2_ < *W*_1_, the value change rate, *a*, of each of the individuals is different, and *a*_2_ ≤ *a*_1_ (see Fig 3). After saving and following the value change of their current wealth, the new wealth values are $\tilde{W_{1}}=W_{1}+sD+a_{1}W_{1}$ and $\tilde{W_{2}}=\rho W_{1}+sD+a_{2}\rho W_{1}$, respectively. The difference between $\tilde{W_{1}}$ and $\tilde{W_{2}}$ is larger than *W*_1_−*W*_2_, but since inequality is a relative measure, the value of $\frac{\tilde{W_{1}}}{\tilde{W_{2}}}$ should be analyzed with respect to $\frac{W_{1}}{W_{2}}=\frac{1}{\rho }$. Substituting $\tilde{W_{1}}$ and $\tilde{W_{2}}$ with the above expressions, we obtain a constraint on *s*, so that the ratio between the individual wealth values is not changed:

$$\begin{array}{c} \frac{\tilde{W_{1}}}{\tilde{W_{2}}}=\frac{W_{1}+sD+a_{1}W_{1}}{\rho W_{1}+sD+a_{2}\rho W_{1}}=\frac{1}{\rho }\Rightarrow s=\frac{\rho W_{1}\left(a_{1}-a_{2}\right)}{\left(1-\rho \right)D}\;. \end{array}$$ (7)

Therefore, when personal savings are larger than $\frac{\rho W_{1}(a_{1}-a_{2})}{(1-\rho )D}$, with the constraint *s* < 1, we obtain $\frac{\tilde{W_{1}}}{\tilde{W_{2}}}<\frac{1}{\rho }$, meaning that the wealth inequality is decreased. For a lower savings rate the relative difference increases and the wealth inequality increases. By randomly sampling individuals within the populations created for the numeric calculations and based on the data for the dependence of *a* on wealth (see Fig 3), in 98.5% of the cases the value of $\frac{\rho W_{1}(a_{1}-a_{2})}{(1-\rho )D}$ was less than 1, with an average value of 0.016, or 1.6%, a clearly realistic savings rate.

This example illustrates that due to the additive effect of income and the multiplicative nature of return, and since the income distribution differs from the wealth distribution, the relative gap between the bottom deciles and top decile becomes narrower, when personal savings are large comparing to the other parameters. This effect would vanish if wealth and income were almost identically distributed and highly correlated.

### The effect of income distribution on wealth inequality

In addition to the substantial effect of personal savings on wealth inequality, the results displayed in Fig 5 shed light on the effect of income tax and income inequality on wealth inequality. In particular, it was suggested that increasing income tax progressively might contribute to lowering wealth inequality [20, 25, 52]. In addition, it follows from Eqs (2) and (5) that increasing the average tax rate reduces the disposable income, hence effectively reduces savings. Moreover, increasing progressive income tax also reduces the income inequality. In short, taxation reduces savings, but redistributes income more equally, and therefore increasing progressive income tax creates a trade-off between the effects of the decreased savings and the decreased income inequality.

The results presented in Fig 5 demonstrate that the inner distribution of income tax has a small effect on the dynamics of wealth inequality. A drastic linear decrease in income inequality to an income Gini index of 0.15 over a period of 23 years, resulted in an insignificant decrease of the share of wealth owned by the top decile from 78.9% to 78.6%. Once again, this small effect is attributed to the imperfect correlation between wealth and income.

In order to test the effect of changing the income Gini index on wealth inequality, we set the Gini index to linearly increase/decrease to values ranging from 0.1 to 0.9 in the years 2008–2030 and calculated the final share of wealth owned by the top 10% of the population. This calculation resulted in an approximate linear relationship between the final wealth inequality and the final income inequality. The obtained range of the final wealth inequality values was relatively narrow. For the extreme Gini index values of 0.1 and 0.9, the final values of the share of wealth owned by the top 10% were 78.6% and 79.3%, respectively. This narrow range indicates that indeed, changing the income distribution has only a small effect on wealth inequality.

**Fig 7:**
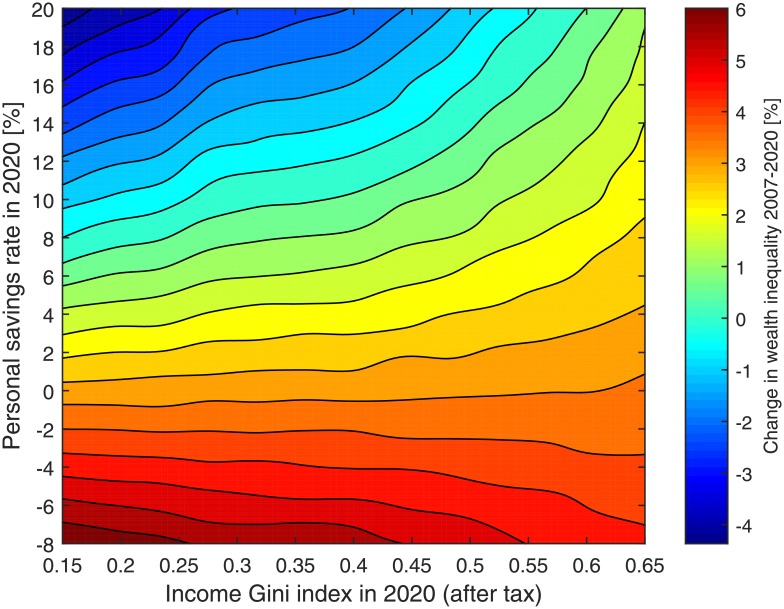
The joint effect of personal savings and income inequality on wealth inequality. This contour plot displays the change in wealth inequality between 2007 and 2020, according to the model results, given that the income inequality Gini index values were linearly changed up to a final value ranging between 0.15 to 0.65 (after tax) and the personal saving rates were linearly changed at the same time up to a final value between -8% to 20%. The other model parameters were extrapolated as done for the predicted results. The curliness of some of the contour is due to statistical error.

Nevertheless, these above results and their implications are valid, as long as the correlation between wealth and income remains low. As income inequality increases, this correlation is likely to increase as well. We tested this argument by considering the model with all parameters taking their historical values between 1930 to 2007 for the US economy. From 2007 to 2020, we considered a linear extrapolation of all the parameters, however this time we let the personal savings and the income Gini index to be controlled at the same time. The latter two parameters were considered as linearly increasing/decreasing from their values at 2007 to varying values running from -8% to 20% for the personal savings and from 0.15 to 0.65 (after-tax) for the income Gini index (*G*). For each final value of savings and income inequality we performed a numerical simulation using the model, according to the described procedure, and this way we calculated the change in wealth inequality between 2007 and 2020. The results of this calculation are presented in Fig 7.

The results demonstrate again that increasing personal savings has a strong effect on reducing wealth inequality, while changing the income Gini index has a mild effect on wealth inequality. However, what is apparent from these results, which could not be deduced from the previous results, is that as income inequality increases, the effect of savings on wealth inequality weakens. Specifically, when the Gini index is above 0.6, the personal savings tend to have only a mild effect on wealth inequality. The reason for this effect is that in such cases, in which income inequality is so high, the correlation between wealth and income becomes much higher than in practice—roughly 0.8, rather than 0.55–0.6 [21, 23]. Income Gini index values higher than 0.6 are very rare, and none of the OECD countries have such a high level of income inequality nowadays [53]. South Africa is the only major country with Gini index of about 0.65 (pre-tax) [54]. However, if the recent income inequality surge will continue in the next few decades, the US economy might reach a point in which wealth and income will be strongly correlated and increasing savings will cease to have a major effect on the distribution of wealth. These results demonstrate the trade-off between the different effects of changing the progressive income tax.

## Worldwide Wealth and Income Inequality

**Fig 8:**
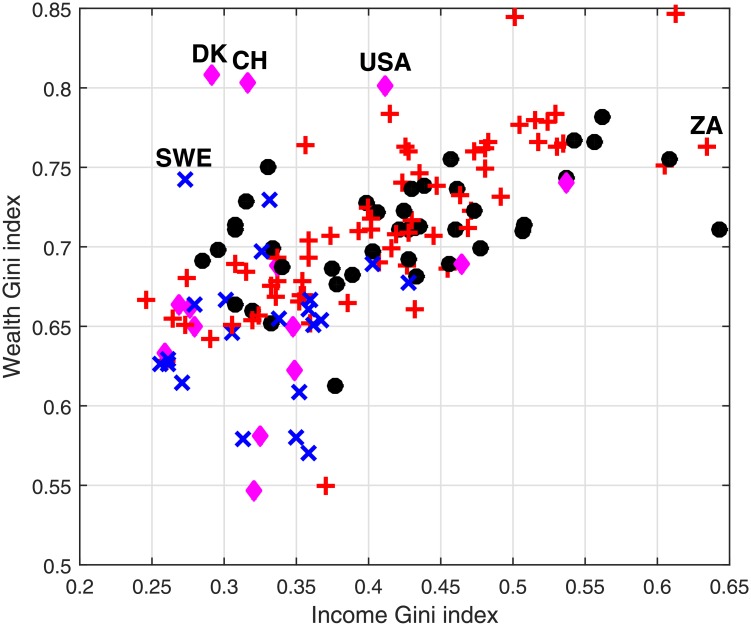
Income and wealth inequality worldwide. The wealth and after-tax income Gini indices for various countries divided according to income levels (GDP per capita in PPP 2000 dollars)—low (black circles), middle (red plus signs), middle-high (blue crosses) and high (magenta diamonds), corresponding to < 2500$, < 12000$, < 25000$ and > 25000$ per year. Sweden (SWE), Denmark (DK), Switzerland (CH), USA (USA) and South-Africa (ZA) are tagged. The data are taken from [15] and [54] and is relevant for the early 2000’s.

The model and the analysis so far were implemented on the US only. However, more general conclusions can be deduced regarding wealth inequality and the relationship between income and wealth inequality. While the importance of personal savings is clear (see Fig 6), the results in Fig 7 indicate that when income inequality is very high compared to wealth inequality, the effect of personal savings tends to be milder. A thorough analysis as done for the US is practically impossible for most countries due to the lack of sufficient data. However, it is possible to discuss the current income and wealth inequality in various countries and apply our conclusions accordingly. This is specifically interesting cross-sectionally between countries belonging to different income levels. We divided 140 countries to 4 income levels according to their GDP per capita (PPP)—low, middle, middle-high and high (< 2500$, < 12000$, < 25000$ and > 25000$, respectively, measured in 2000 PPP dollars) and for each country we considered its after-tax income Gini index and wealth Gini index (data were taken from [15] and [54]). These data is presented in Fig 8.

There is an obvious positive correlation between wealth and income inequality. However, it is a characteristic of mainly poor countries. The correlation between the Gini indices for the middle-high and high levels of income is 0.23, while for the low and middle income countries it is 0.68. This indicates that our conclusions regarding the role of savings will probably have less importance when poorer economies are considered. When the difference between these inequalities is high, for example, such as in the US, Sweden, Switzerland and Denmark, personal savings will likely have a large effect on wealth inequality. This effect will not be as strong in countries such as South Africa and China, where income inequality have a larger role in the creation of wealth inequality. Furthermore, in such countries, progressive income taxation may have a significant impact on both income and wealth inequality. These observations make sense, since richer countries are characterized usually by a larger and more dominant financial sector, which is sometimes thought to impose high levels of wealth inequality [6, 10]. These results also highlight the need for the distinction between wealth and income inequalities and indicate that the dynamics of wealth inequality in low income countries are fundamentally different from these dynamics in rich countries.

## Discussion

We devised a reliable model for the dynamics of wealth inequality. Using the historical values to estimate the model parameters, we were able to reproduce the historical behavior of wealth inequality in the United States during 1930–2010. The model provides a valuable tool to estimate the effect of income tax and personal savings on wealth inequality. We note that the model does not incorporate any free parameters that need to be adjusted, and all of our assumptions are made using realistic estimations of the parameters, based on real data.

Following the simulation results it can be concluded that the personal savings fraction is highly influential on wealth inequality. This conclusion indicates that in the early 1980s the United States economy gradually switched from being income-dominated to being capital-dominated [6, 9, 11]. In particular, the obtained results imply that the wealth inequality surge in the United States since the 1980s is, to a large extent, due to the major decrease in personal savings in the same period, as suggested by previous studies [7, 12–14]. In addition, the observed behaviors of wealth inequality as well as of personal savings in the past few decades are almost universal. Wealth inequality in most developed countries is gradually increasing since the 1980s [6]. This increase is accompanied by a decrease in personal savings, especially in the US and Japan [6, 11, 55]. We note again, that this effect does not imply anything on social mobility, but only accounts for the possibility of mainly middle and higher wealth deciles to narrow the relative gap between their wealth and the wealth of the highest wealth deciles. It cannot be deduced from our results that poor individuals would become very rich if they save a large fraction of their income.

Mathematically, increasing the average tax rate reduces the disposable income, hence effectively reduces savings. However, the relatively limited range of average income tax rates makes its effect on wealth inequality relatively low, in practice. Therefore, it can be concluded that progressive income tax is likely to be an impractical measure for affecting wealth distribution, as suggested previously [20, 25, 36, 52]. This non-trivial effect of income on wealth inequality is originated from the imperfect correlation existing between income and wealth [18, 21, 23, 24]. When income tax is increased, the top earners, who are not necessarily the wealthiest individuals in the population, have a larger difficulty of accumulating wealth, with respect to the wealthiest. On the other hand, it barely affects the wealthiest individuals. Therefore, such an increase might even deepen the wealth gap. When decreasing income tax, the opposite effect will therefore occur. In addition, progressive taxation, which might have a significant effect on the distribution of income, will have a small effect on wealth inequality. Therefore, we conclude that affecting income inequality or changing the average income tax in order to effect wealth inequality are likely to be ineffective means. An evidence for this can be found in the economies of Denmark and Sweden, two of the most equal countries in terms of income in the world, with a high tax burden, in which wealth inequality is among the highest within the OECD countries [53].

Following our results, we predict that given an ongoing substantial increase in income inequality, which would also lead to an increase of the correlation between wealth and income, personal savings will likely have a weaker effect on wealth inequality. By using the model for making estimations of the future wealth inequality in the US, we have also shown that wealth inequality is likely to continue increasing in the following decades. However, a substantial increase in personal savings might cause the reduction of wealth inequality within a few years. Any realistic change in income tax will have no significant effect on wealth inequality. We also note that low to middle income countries are characterized by a stronger relationship between wealth and income when compared to richer economies. Therefore, personal savings are likely to have a milder effect on wealth inequality in those countries, as illustrated for in Fig 7.

The analysis of inequality within the top wealth fractiles of the population, such as the top 1%, 0.1% or 0.01% fractiles, using the presented model, is currently problematic. There are two main difficulties which should be overcome for this purpose:

1. Wealth accumulation processes characterizing the top fractiles are governed by capital income and the change in the value of capital, and much less on labor income or on savings. Therefore, a more intricate description of mechanisms that affect capital income should be introduced in order to model these effects.
2. The model parameters considered, were taken in the resolution of the population income and wealth deciles. Therefore, if higher fractiles are considered, a higher resolution in those parameters is required. Specifically, the dependence of savings and of the capital value change rate on income and wealth percentiles is different from the dependence on deciles. Therefore, one cannot obtain a reliable result for the share of wealth of higher fractiles, based on the decile dependent parameters only. We also note that such high resolution data are very difficult to obtain with reliable precision.

This limitation of the model joins additional limitations of the current version of the model. Economic mobility, which might have a small effect on wealth inequality in the US, is not fully addressed in the proposed model. In some economies, in particular young economies, or such that undergo significant reforms and deep social changes, mobility might play a significant role. Inheritance and inheritance tax policies are not incorporated explicitly in the model, as we also don’t discuss population growth. These might have substantial effects on the wealth inequality when the population growth rates are very high (in Africa and in the middle east, for example), or when the inheritance tax rates are very high. In most western countries, the population growth rates are very low, and inheritance taxes were abolished or are very low.

Reliable modeling of wealth inequality dynamics with models such as this one can serve regulators and policy makers in their efforts to control wealth inequality. However, while describing the mechanisms that might contribute to lowering wealth inequality, such as increasing the personal savings, the implications of such processes on other aspects of the economy were not considered. Such implications should be taken into account when policy and regulation are considered. Notably, substantially increasing personal savings might have a major positive effect on wealth inequality. However, its marginal effect is reduced as the savings rate increases and a very high rate might also limit the GDP growth [56, 57]. Nonetheless, we note that the effects of savings on economic growth are intricate and under debate within the economic community, generally relating increasing savings to an increase in economic output in the long run, with less obvious short term effects [58–62].

More research should be carried out to establish the above: further validation of the model by considering additional economies; incorporating additional factors in the model and quantitative estimation of the parameter values leading to the decrease of wealth inequality; analyzing the unique characteristics of the 2008 financial crisis in terms of wealth distribution and their effect on the model; considering the effect of the shadow economy on actual inequality, since taking it into account may change the wealth and income distributions; determining the effect of inheritance, population growth and economic mobility on wealth inequality.

## Supporting Information

S1 Fig The wealth-income correlation. The model results for the correlation between the wealth and the disposable income for the nominal calculation of the wealth distribution in the US during 1930–2010. The model parameters are the same as used for the calculation presented in Fig 4.
(PDF)

S2 Fig The effect of wealth dependent value change of capital on wealth inequality. Left panel: The model results for the top 10% share of wealth in the United States during 1930–2010, given different dependencies of the capital value change on wealth. The nominal maximal ratio, according to Wolff is 2.2 (red). The other values considered were (in dotted curves) 1.8 (magenta), 2.2 (cyan), 2.4 (black) and 2.6 (green). The data for the historical behavior of the wealth inequality (blue) were taken from Saez and Zucman [20]. Right panel: The dependence of the Pearson correlation between the historical behavior of the wealth inequality in the US and the model results in the maximal ratio value.
(PDF)

S3 Fig The model results for the top 10% share of wealth in the United States during 1930–2010 with constant income Gini index. The results produced by implementing the model with a constant Gini index (green) and taking into account the historical Gini index (red). The rest of the parameters were considered with their historical values. The data for the historical behavior of the wealth inequality (blue) were taken from Saez and Zucman [20].
(PDF)

S4 Fig The model results for the historical wealth inequality measures in the United States during 1930–2010 with statistical error-bars. The results produced by implementing the model (red) were calculated using the historical data for the various parameters [11, 20]. The historical data (blue) were taken from Saez and Zucman [20]. The results are given for the top 10% share of wealth (left) and for the reconstructed historical Gini index (right), based on Saez and Zucman [20]. The error-bars signify one standard deviation based on the statistical spread of the results due to the random sampling of the initial values of wealth and income.
(PDF)
