# Supplementary figures and images for: The Dynamics of Wealth Inequality and the Effect of Income Distribution
Source: PLoS One. 2016 Apr 22;11(4):e0154196. doi: 10.1371/journal.pone.0154196 (PMC4841595; doi:10.1371/journal.pone.0154196)

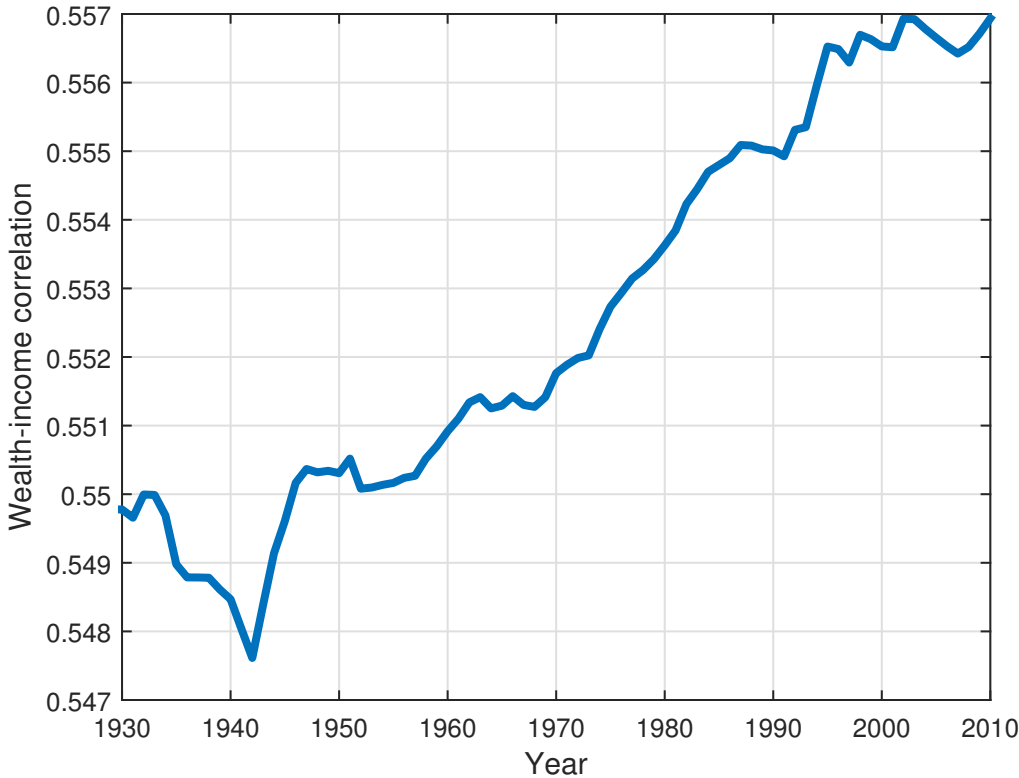

Supplement: S1 Fig — The model results for the correlation between the wealth and the disposable income for the nominal calculation of the wealth distribution in the US during 1930–2010. The model parameters are the same as used for the calculation presented in Fig 4. (PDF) [file pone.0154196.s001.pdf]

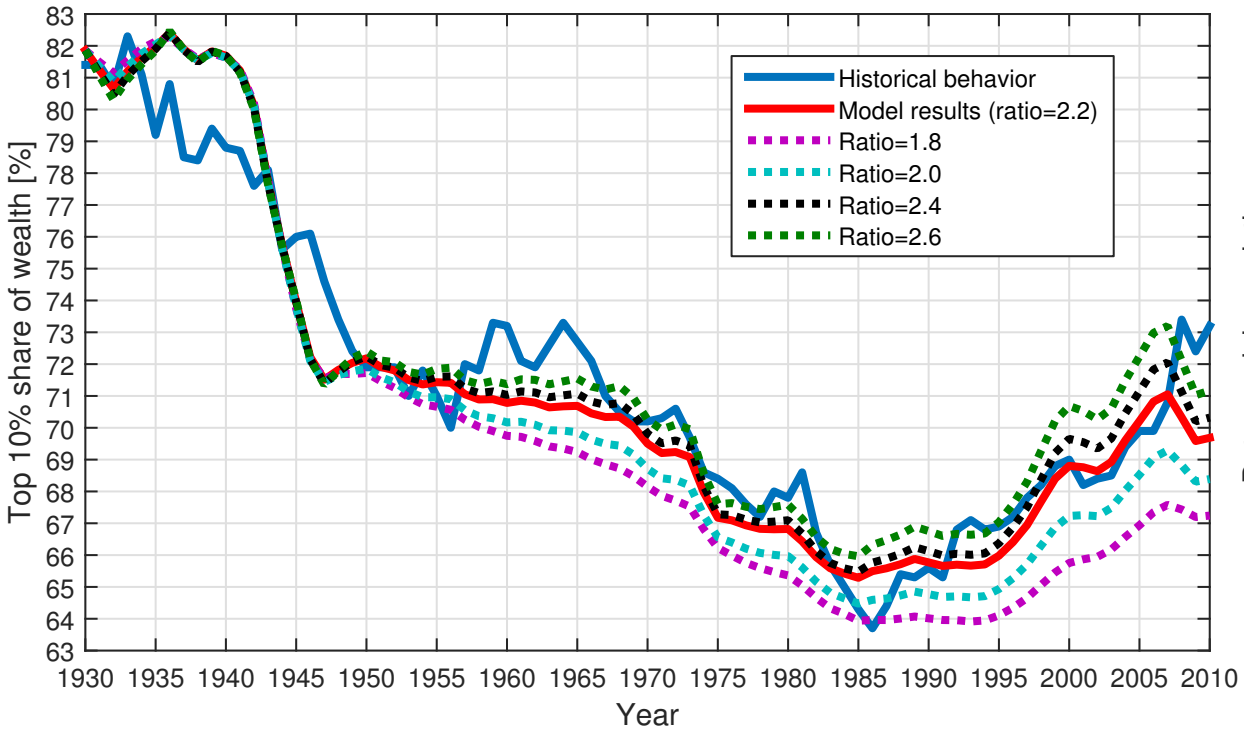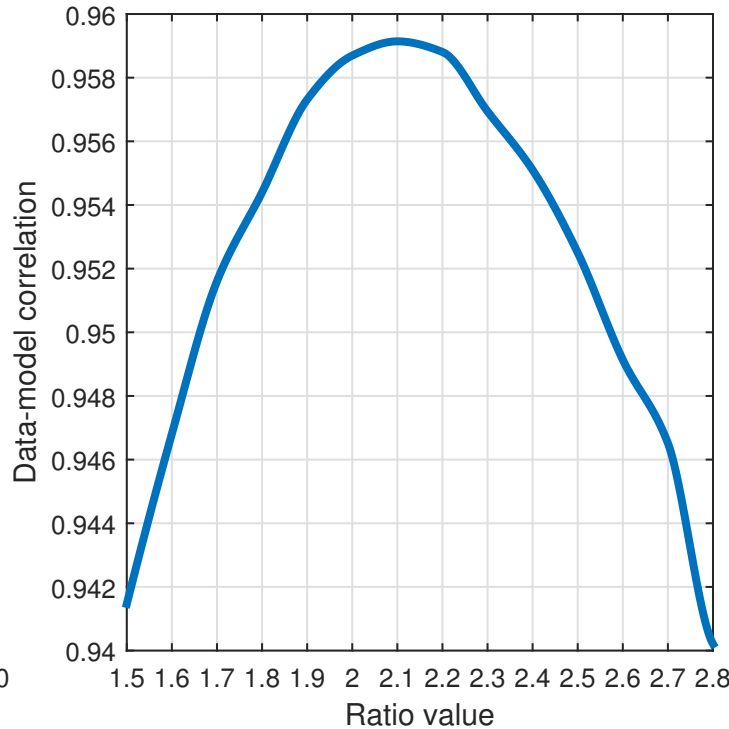

Supplement: S2 Fig — Left panel: The model results for the top 10% share of wealth in the United States during 1930–2010, given different dependencies of the capital value change on wealth. The nominal maximal ratio, according to Wolff is 2.2 (red). The other values considered were (in dotted curves) 1.8 (magenta), 2.2 (cyan), 2.4 (black) and 2.6 (green). The data for the historical behavior of the wealth inequality (blue) were taken from Saez and Zucman [20]. Right panel: The dependence of the Pearson correlation between the historical behavior of the wealth inequality in the US and the model results in the maximal ratio value. (PDF) [file pone.0154196.s002.pdf]

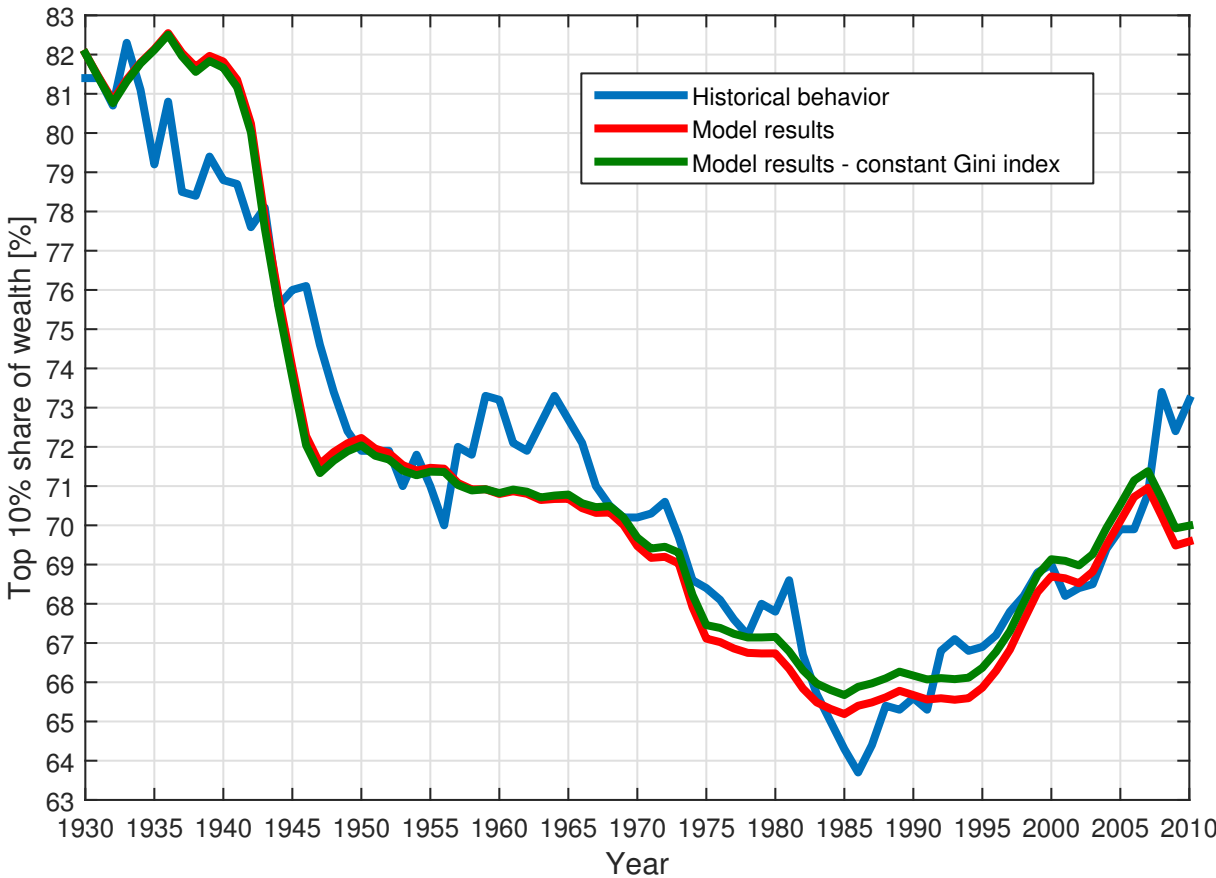

Supplement: S3 Fig — The results produced by implementing the model with a constant Gini index (green) and taking into account the historical Gini index (red). The rest of the parameters were considered with their historical values. The data for the historical behavior of the wealth inequality (blue) were taken from Saez and Zucman [20]. (PDF) [file pone.0154196.s003.pdf]

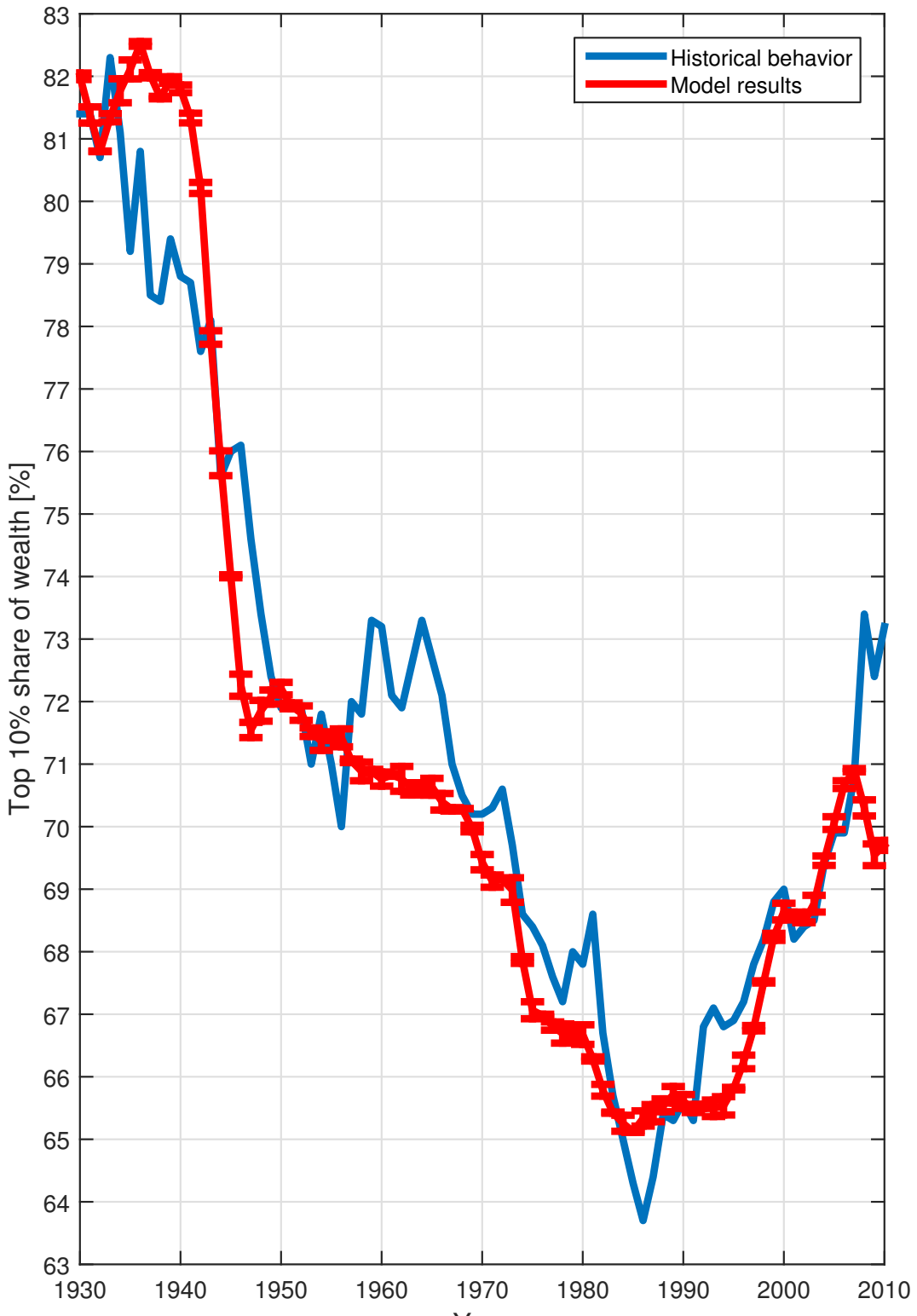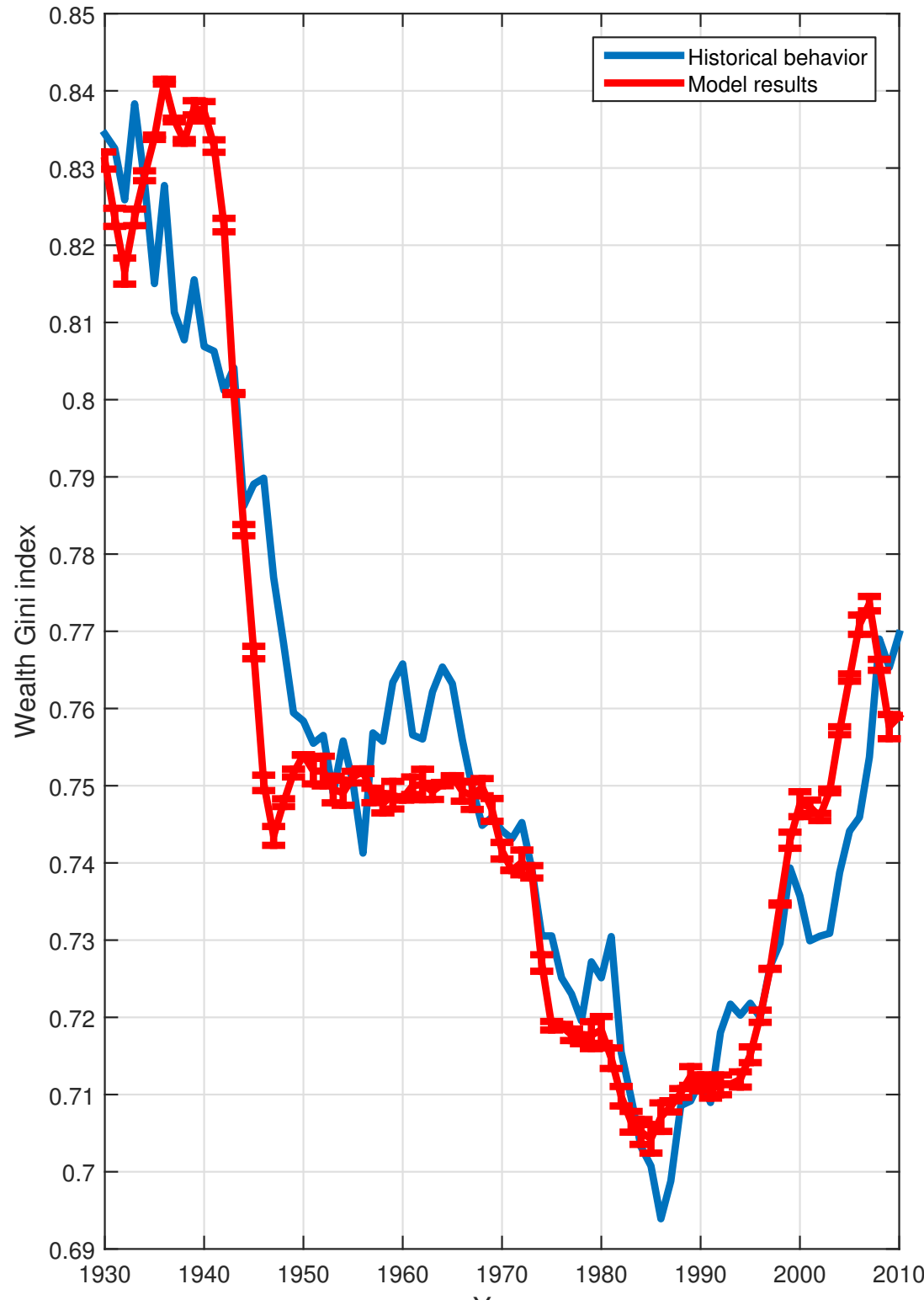

Supplement: S4 Fig — The results produced by implementing the model (red) were calculated using the historical data for the various parameters [11, 20]. The historical data (blue) were taken from Saez and Zucman [20]. The results are given for the top 10% share of wealth (left) and for the reconstructed historical Gini index (right), based on Saez and Zucman [20]. The error-bars signify one standard deviation based on the statistical spread of the results due to the random sampling of the initial values of wealth and income. (PDF) [file pone.0154196.s004.pdf]
